# Supplementary material for: Pandemic lifeworlds: A segmentation analysis of public responsiveness to official communication about Covid-19 in England
Source: PLoS One. 2024 Jan 31;19(1):e0296049. doi: 10.1371/journal.pone.0296049 (PMC10830050; doi:10.1371/journal.pone.0296049)

Appendix A: Multidimensional Scaling for Preliminary Assessment of Segment Interpretability

Figure 1 arranges the seven segments, as comprised in the initial data base with unrefined factors, in multi-dimensional space. The interpretability of the segments as arranged in multidimensional space encouraged development of the final segmentation scheme. The segments more compliant with public health recommendations are to the left and those that are less compliant to the right. The higher on the figure they are, the greater their feelings of control over their lives. The lower they are in the figure the more powerless they feel.


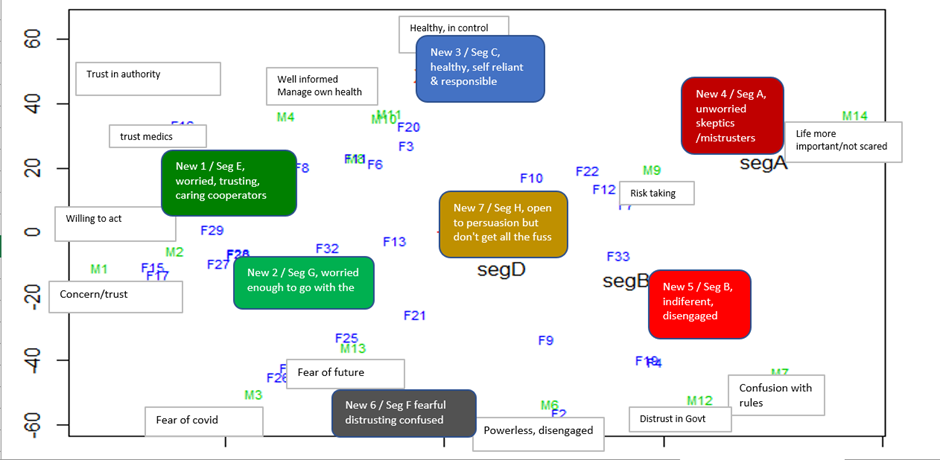


Figure 2 illustrates five different axes that help to further differentiate our segments. In addition to high control versus powerlessness and high versus low compliance, we see high versus low trust, fear, and feelings of being well-informed regarding public health guidance. These distinctions, based on the final segmentation assignments, are detailed in the profile descriptions; however, by visualizing them graphically in relation to one another helps encapsulate the complexity inherent in these segment descriptions.


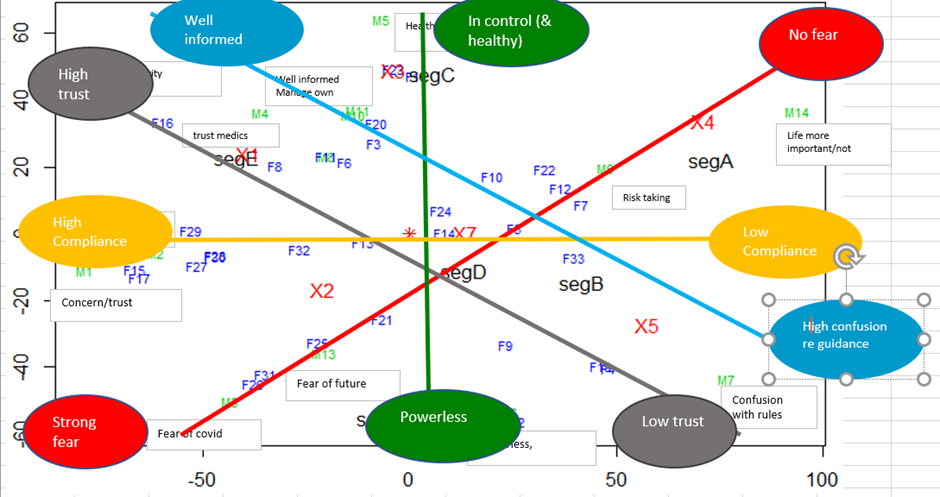

Supplement: S1 Appendix — (ZIP) [file pone.0296049.s001.zip › Appendix 1 (002).docx]
