# Supplementary material for: Pandemic lifeworlds: A segmentation analysis of public responsiveness to official communication about Covid-19 in England
Source: PLoS One. 2024 Jan 31;19(1):e0296049. doi: 10.1371/journal.pone.0296049 (PMC10830050; doi:10.1371/journal.pone.0296049)
Supplement: Attachment — Submitted filename: Review report.docx [file pone.0296049.s003.docx]

**Review report of the article “Pandemic life worlds a segmentation analysis of public responsiveness to official communication about covid-19 in England”**

**By**

**Stephen Coleman, Michael D Slater, Phil Wright, Oliver Wright, Lauren Skardon, Gillian Hayes**

This article presents the findings of a comprehensive audience segmentation study conducted in England, sponsored by the United Kingdom Health Security Agency (UKHSA) and executed through a collaborative effort between market research professionals and academic experts. The study employs various methods and reports results that are compared with analogous audience segmentation endeavours worldwide in the context of the COVID-19 pandemic. The article briefly outlines multiple communication strategies tailored to distinct audience segments and underscores the benefits of detailed segmentation descriptions in enhancing our understanding of public health communication audiences.

This study offers valuable insights for future research in this domain and, therefore, I recommend its publication in the PLOS ONE journal, contingent upon the authors addressing the following comments:

1. Abstract Revision: It is advisable for the authors to omit the headings such as "Background," "Methods," "Results," and "Conclusion" in the Abstract section, as these aspects are already comprehensively covered in subsequent sections of the article.
2. Citation Enhancement: To provide readers with access to the latest developments in this field and to complete the reference section, I suggest that the authors include the following references in their manuscript:
a) These revisions will enhance the clarity and conciseness of the article, making it a valuable addition to the PLOS ONE journal.
b) Tiwari, S. (2022). Impact of COVID -19 Era on Supply Chain Management and Logistics of Flipkart Company. VEETHIKA-An International Interdisciplinary Research Journal, 8(4), 25-27.
c) Gupta, S., & Tiwari, S. (2023). New Technological Advancements and Its Impact on Healthcare System. *VEETHIKA-An International Interdisciplinary Research Journal*, *9*(1), 27-32.
d) Suresh K.P., Kumar M., Shinli V. 2022. Tribal Healthcare System in Kerala during the Pandemic, Dera Natung Government College Research Journal, 7, 83-93. DOI: <https://doi.org/10.56405/dngcrj.2022.07.01.09>
e) Siriwardhanaa W.S.N, Rathnayakab R.M.S.S. 2022. Awareness of Counseling Psychology and the Significance of Counseling Service for the Graduate Studies, Dera Natung Government College Research Journal, 7, 70-75. DOI: <https://doi.org/10.56405/dngcrj.2022.07.01.07>
f) Bosumatary S. 2019. Social Inequality and Health: A Study of Tribes in Assam, Dera Natung Government College Research Journal, 4, 7-14. DOI: <https://doi.org/10.56405/dngcrj.2019.04.01.02>

These revisions will enhance the clarity and conciseness of the article, making it a valuable addition to the PLOS ONE journal.
